# Entry of ZSWIM4 to the nucleus is crucial for its inhibition of KIT and BMAL1 in gastrointestinal stromal tumors

**DOI:** 10.1186/s13578-024-01271-z

**Published:** 2024-06-29

**Authors:** Xu Cao, Jinhai Tian, Man Yee Cheung, Liangying Zhang, Zimei Liu, Zongying Jiang, Shaoting Zhang, Kun Xiao, Sien Zhao, Ming Wang, Feng Ding, Shujing Li, Lijun Ma, Hui Zhao, Jianmin Sun

**Affiliations:** 1 NHC Key Laboratory of Metabolic Cardiovascular Diseases Research, School of Basic Medical Sciences, Ningxia Medical University, Yinchuan, China; 2 Key Laboratory for Regenerative Medicine, Ministry of Education, School of Biomedical Sciences, Faculty of Medicine, The Chinese University of Hong Kong, Hong Kong SAR, China; 3 Department of Oncology, School of Medicine, Tongren Hospital, Shanghai Jiao Tong University, Shanghai, China; 4 The General Hospital of Ningxia Medical University, Yinchuan, China; 5 Kunming Institute of Zoology - The Chinese University of Hong Kong (KIZ-CUHK) Joint Laboratory of Bioresources and Molecular Research of Common Diseases, The Chinese University of Hong Kong, Hong Kong SAR, China; 6 Hong Kong Branch of CAS Center for Excellence in Animal Evolution and Genetics, The Chinese University of Hong Kong, Hong Kong SAR, China

**Keywords:** GISTs, ZSWIM4, KIT, BMAL1, Signaling

## Abstract

**Background:**

Zinc finger SWIM-type containing 4 (ZSWIM4) is a zinc finger protein with its function largely uncharacterized. In this study, we aimed to investigate the role of ZSWIM4 in gastrointestinal stromal tumors (GISTs).

**Results:**

We found that ZSWIM4 expression is inhibited by the predominantly mutated protein KIT in GISTs, while conversely, ZSWIM4 inhibits KIT expression and downstream signaling. Consistent with the observation, ZSWIM4 inhibited GIST cell survival and proliferation in vitro. RNA sequencing of GISTs from KIT^V558A/WT^ mice and KIT^V558A/WT^/ZSWIM4^−/−^ mice showed that loss of ZSWIM4 expression increases the expression of circadian clock pathway member BMAL1 which contributes to GIST cell survival and proliferation. In addition, we found that KIT signaling increases the distribution of ZSWIM4 in the nucleus of GIST cells, and which is important for its inhibition of KIT and BMAL1. In agreement with the results in vitro, the in vivo studies showed that ZSWIM4 deficiency increases the tumorigenesis of GISTs in KIT^V558A/WT^ mice.

**Conclusions:**

Taken together, our results revealed that the entry of ZSWIM4 to the nucleus is important for its inhibition of KIT and BMAL1, ultimately attenuating GIST tumorigenesis. The results provide a novel insight in the understanding of signal transduction in GISTs and lay strong theoretical basis for the advancement of GIST treatment.

**Supplementary Information:**

The online version contains supplementary material available at 10.1186/s13578-024-01271-z.

## Introduction

Gastrointestinal stromal tumors (GISTs) are mesenchymal tumors that predominantly occur in the stomach and small intestine, they can be found in the other parts of the digestive tract but are uncommon [1]. GISTs are considered to originate from the interstitial cells of Cajal (ICC) that act as the pacemakers of the digestive tract [2] although smooth muscle cells can be transformed into GIST cells as well [3]. At present, the treatment of GISTs relies on surgery and targeted therapy against KIT. Surgery is the first choice for low-risk GISTs, while adjuvant therapy using KIT inhibitors is necessary for high-risk tumors [4, 5].

Genomic sequencing has identified genetic mutations in GISTs. Among all mutations, KIT mutations account for 70–80% of all tumors, and PDGFRA mutations account for around 10% of GISTs [6–9]. Based on that, small molecule inhibitors of KIT have been developed and applied in the treatment of GISTs in clinic. Imatinib is the first approved small-molecule inhibitor of KIT in the treatment of GISTs [10], and it has dramatically improved treatment outcomes. However, GISTs can acquire drug-resistant secondary mutations in KIT [11–13] or activate alternative signaling pathways [14–16] to circumvent the inhibition of KIT after the initial response to imatinib, leading to tumor relapse. Although sunitinib, regorafenib, and ripretinib can be used as the second-, third-, or fourth-line therapeutic drugs of GISTs after the failure of imatinib treatment, they can only increase the patient survival for a few months respectively [17–19].

KIT belongs to type III receptor tyrosine kinase together with PDGFRA, FLT3, and CSF1R [20, 21]. As a transmembrane protein, KIT has an extracellular region with five immunoglobulin-like domains, a transmembrane region, and an intracellular region with the kinase domain separated by a kinase insert. After binding with its ligand, stem cell factor (SCF), KIT is activated and phosphorylated on its tyrosine residues in the intracellular region, which further recruits and activates downstream signaling pathways such as RAS/RAF/MEK/ERK and PI3 kinase/AKT signaling pathways, mediating cell survival, proliferation and differentiation. Unlike the wild-type KIT whose activation needs ligand binding, the gain-of-function mutations of KIT that occur in GISTs and other malignancies such as mastocytosis confer the ligand-independent activation to the receptor, leading to uncontrolled signaling and eventually cell transformation [21, 22].

Zinc finger SWIM-type containing 4 (ZSWIM4) is a zinc finger protein whose function has been largely unexplored. Our recent results identified that ZSWIM4 forms a complex with the Cul2-RING ubiquitin ligases, ELOB and ELOC, facilitating the ubiquitination and subsequent degradation of SMAD1 during embryonic development [23]. In this study, we found that ZSWIM4 expression is downregulated in GISTs. Notably, KIT and ZSWIM4 appear to mutually repress each other's expression. Furthermore, we found that KIT signaling increases the nuclear translocation of ZSWIM4, which is crucial for its role in inhibiting both KIT signaling and the circadian clock pathway component BMAL1, ultimately leading to a reduction in GIST tumorigenesis.

## Materials and methods

### Reagents, antibodies, and plasmids

The anti‐KIT antibody was purified as previously described [24] by Jihua Biotechnology. The anti‐pY antibody 4G10 and chemiluminescent HRP substrate were purchased from Millipore (Billerica, MA). AKT, ERK, phospho‐ERK (T202/Y204), BMAL1, and HRP conjugated β-actin antibodies were purchased from Santa Cruz Biotechnology (Dallas, TX). The phospho‐AKT (S473) antibody was purchased from Cell Signaling Technology (Danvers, MA). The ZSWIM4 antibody was purchased from Biorbyt (Durham, NC). FLAG antibody was purchased from Sigma-Aldrich (St. Louis, MO). The Lamin B1 antibody was purchased from Abmart (Shanghai, China). HRP conjugated goat anti‐mouse IgG antibody, and HRP conjugated goat anti‐rabbit IgG antibody were purchased from Bioss Antibodies (Beijing, China). YF 594 conjugated goat anti‐rabbit IgG and YF 488 conjugated goat anti‐mouse IgG were purchased from UE Landy (Suzhou, China). Fluor 594 conjugated goat anti‐mouse IgG was purchased from Affinity Biosciences (Liyang, China). ZSWIM4 siRNAs and BMAL1 siRNAs were synthesized by Sheweisi Biotech (Tianjin, China). KIT siRNAs were synthesized by Genepharma (Shanghai, China). Nuclear and cytoplasmic protein extraction kit was purchased from Beyotime (Shanghai, China). Annexin V‐PE apoptosis detection kit was purchased from BD Biociences (San Jose, CA). The cell cycle detection kit and MTT Kit were purchased from Keygen Biotech (Nanjing,China). Imatinib was purchased from MedChemExpress (Monmouth Junction, NJ). Lipofectamine 2000 was purchased from Thermo Fisher Scientific (Waltham, MA). PrimeScript RTase and SYBR^®^ Premix Ex Taq^™^ kit were purchased from Takara (Dalian, China). ZSWIM4 expressing plasmid was constructed previously [23]. ZSWIM4^MUT^ which lacks 45 amino acids in the N-terminal was amplified by PCR and inserted into pcDNA3.1 + . BMAL1 cDNA with FLAG tag at the C-terminal was synthesized and inserted into pcDNA3.1 + .

### Cell culture

GIST‐T1 cells (STR profiled, December 2023) were grown in RPMI 1640 medium supplemented with 10% fetal bovine serum (FBS), 100 units/mL penicillin, and 100 μg/mL streptomycin.

### Plasmid and siRNA transfection

Plasmids and siRNAs (50 nM) were transfected into GIST‐T1 cells using lipofectamine 2000 according to the manufacturer's instructions. The siRNAs are listed in Table 1.

**Table 1:** siRNAs

Target genes	Sequences
Human KIT‐siRNA1	GGAUGGCACCUGAAAGCAUTT
Human KIT‐siRNA2	GCAACUGCUUAUGGCUUAATT
Human ZSWIM4‐siRNA1	GCAGUGAACGGGAAAUAUGUATT
Human ZSWIM4‐siRNA2	ACAGCAUUUCCUGCUGGAGAATT
Human BMAL1‐siRNA1	CCGAGGGAAGAUACUCUUUTT
Human BMAL1‐siRNA2	CCUGCAUCCUAAAGAUAUUTT

### Cell lysis and western blot

GIST-T1 cells were washed twice with PBS and incubated in RPMI 1640 medium without serum in the cell incubator for 4 h at 37 °C. After washing with ice‐cold PBS, the cells were lysed on ice in a lysis buffer containing 1% Triton X‐100, 25 mM Tris, pH 7.5, 150 mM NaCl, 5 mM EDTA, 10% glycerol, 2 mg/mL aprotinin, 1 mM Na_3_VO_4_, and 1 mM phenylmethylsulfonyl fluoride. Similarly, GIST tissues from mice were lysed on ice in the same lysis buffer. The lysates were centrifuged at 12,000 rpm for 10 min at 4 °C, and the supernatants were boiled in SDS-PAGE loading buffer for 5 min, separated by SDS‐PAGE, and transferred to PVDF membranes (Millipore). The membranes were blocked in PBS with 0.2% tween‐20 (PBST) overnight at 4℃. After incubation with indicated primary antibody overnight at 4 °C and washing with PBST, the membranes were incubated with HRP conjugated secondary antibody for 2 h at room temperature, washed with PBST, and developed with chemiluminescent HRP substrate.

### RNA extraction and qRT‐PCR

The total RNAs were purified from cultured cells or GIST tissues using the RNA simple total RNA kit (Tiangen) according to the manufacturer's instruction, and reverse transcribed into cDNA using a reverse transcription kit (Takara). Real-time PCR was run using a two-step real-time reverse transcription PCR kit (Takara) following the program: 95 °C for 30 s, followed by 44 cycles of 95 °C for 5 s, 60 °C for 34 s. RPL19 was used as control, the relative expression of the target genes was calculated using the 2-∆∆Ct method. The primers are listed in Table 2.

**Table 2:** Primers for qRT-PCR analysis

Target genes	Forward primers	Reverse primers
Human KIT	GCGTTCTGCTCCTACTGCTTCG	TGGATGGATGGTGGAGACGGTT
Human ZSWIM4	AAACCAGAGGAAAGGGCAGG	CCTCTTCCTCCGAGCCTGG
Human BMAL1	GAAGACAACGAACCAGACAATGAGG	GCGTGCCGAGAAACATATTCCATAG
Human NPAS2	GATGTTGGAGGCATTAGATGGCTTC	CCAAGGAGAGGCGTGATACTGTC
Human PER3	CCTACTGCCACTGTTCTGTCCAC	TGCTGTCGCTGCTTCCTGATG
Human CIART	GGAGACCAGTCTAAACACCCAAGG	ACGACCCATCTTCAGCCCATTG
Human NR1D1	CTCATCTTCCTCGTCGTCATCCTC	CACAGTAACACCATGCCATTCAGC
Human NR1D2	GCGAAGGCTGTAAGGGTTTCTTTC	GCGACATTGCTGACATCTGTTCC
Human TEF	GGAGTACATGGACCTGGATGAGTTC	TGGAGGAGGATGGTGGGGATG
Human DBP	AGACCTTTGACCCTCGAAGACATC	CTTGGCTGCCTCGTTGTTCTTG
Human RPL19	ACATGGGCATAGGTAAGCGGAAG	TTCACCTTCAGGTACAGGCTGTG
Mouse KIT	GATCTGCTCTGCGTCCTGTTGG	AACTCTGATTGTGCTGGATGGATGG
Mouse ZSWIM4	AGCCCTGACCCTGTGTGAGAAG	CAGTGGATGCTTCGGATGATGAGAG
Mouse BMAL1	ACGAAGACAATGAGCCAGACAACG	TGTGGAACCATGTGCGAGTGC
Mouse NPAS2	CAGCACAGGCACAGCAGCAG	AAGGTGGAGAGAAGGAGCGAGTC
Mouse PER3	CCTCAGAAGAAGCCAAGCCAATCC	AGTGCGGCGGTGGACAGG
Mouse CIART	GTTGCATCCTATGTCCGCCTGTC	TGGCTAGTCATCTGTGGCTCTGG
Mouse NR1D1	CTTCCTCCTACCCGCCTACCTG	TGTTGCCTTGCCGTAGACTGTTG
Mouse NR1D2	TCCTCGTCCTCGTCTGTTCCATC	GGACAATCTGTGCGGTCACTCTTC
Mouse TEF	GGCTCCTTCCCTCTGGTCCTG	CATGGTACTGGCTGCTGCTGAC
Mouse DBP	AGGCTTGACATCTAGGGACACACC	GGAATGCTTGACAGGGCGAGATC
Mouse RPL19	CCAAGGAAGCACGAAAGC	CAAGAGGGCAACAGACAAAG

### Immunohistochemistry

The study was approved by the Ethics Committee of Ningxia Medical University. 31 cases of GISTs and adjacent tissues were collected by the General Hospital of Ningxia Medical University from 2006 to 2021. The immunohistochemistry was run as previously described [25].

### Cell survival, proliferation, and cell cycle assay

GIST-T1 cells were washed with PBS and digested with 0.05% trypsin, after seeded in 6-well or 96-well plates, and grown in the cell incubator for 72 h, the cells were stained using the PE-Annexin V cell apoptosis detection kit, and apoptotic cells were quantified by flow cytometry. Cell proliferation was evaluated using the MTT kit according to the manufacturer's instructions. For cell cycle assay, cells were stained with propidium iodide (PI), and the cell cycle was analyzed by flow cytometry.

### Immunofluorescence

Paraffin embedded clinical GIST tissue or murine GIST tissue sections were consecutively treated with dimethylbenzene, anhydrous ethanol, 95% ethanol, 85% ethanol, 75% ethanol, 50% ethanol, distilled water, and PBS. The sections were then boiled in sodium citrate solution (pH 6.0) for 5 min and washed with PBS. GIST-T1 cells grown on 35 mm glass bottom cell culture dishes were fixed with formaldehyde and washed with PBS. Both tissue sections and cells were treated with 0.1% triton x-100 at room temperature for 10 min, followed by treatment with 3% H_2_O_2_ solution for 15 min to quench the endogenous peroxidase activity. Subsequently, the tissue sections and cells were incubated with goat serum at 37 ℃ for 20 min, and indicated primary antibodies at 4 ℃ overnight. After incubation with corresponding secondary antibodies that are conjugated with fluorescent dyes at 37 ℃ for 1 h, and staining with DAPI at room temperature for 10 min, the tissue sections and cells were observed under Zeiss laser confocal microscope.

### Animal experiments

The study was approved by the Ethics Committee of Ningxia Medical University. Guide for the Care and Use of Laboratory Animals, and animal care was in accordance with institution guidelines were followed. The conditional ZSWIM4 knockout mice were constructed by Biocytogen Pharmaceuticals Technology Company of China. Briefly, to insert the floxP fragment into intron 1 and intron 2 of murine ZSWIM4 gene loci, two sgRNAs (Table 3) targeting intron 1 and intron 2 were respectively ligated to pT7-sgRNA-2G and the sequences were confirmed by DNA sequencing. After transcription, the sgRNAs were injected into zygotes of C57BL/6N mouse strain background and the founders were genotyped by PCR product sequencing of the region that spams the inserted floxP. The founders were crossed with wild-type C57BL/6N mice and further inbred to construct ZSWIM4^flox/flox^ mice.

**Table 3:** sgRNAs

Target sequences	sgRNAs
GTGAGAAAGACCGAGGACGTGGG	CTATTTCTAGCTCTAAAACACGTCCTCGGTCTTTCTCACCTATAGTGAGTCGTATTA
TCGCAGATGCCCTCTGTGTGAGG	CTATTTCTAGCTCTAAAACCACACAGAGGGCATCTGCCTATAGTGAGTCGTATTA

C57BL/6 J E2a-Cre mice were provided by Cyagen Bioscience Company. ZSWIM4^flox/flox^ mice were crossed with E2a-Cre mice to induce the cleavage of exon 2 of ZSWIM4 between the floxP sites in the genome to generate global ZSWIM4 knockout mice. After PCR analysis of DNA, the heterozygous ZSWIM4^−/+^ mice were then crossed with wild type C57BL/6 J mice to remove the DNA recombinase Cre element in the chromosome. ZSWIM4^−/−^ mice were established by inbreeding of the heterozygous ZSWIM4^−/+^ mice.

Mice carrying germline KIT/V558A mutation were constructed as previously described [26]. KIT^V558A/WT^/ZSWIM4^−/−^ mice were established by crossing KIT^V558A/WT^ mice and ZSWIM4^−/−^ mice. 8-week-old KIT^V558A/WT^ mice and KIT^V558A/WT^/ZSWIM4^−/−^ mice were treated with imatinib (50 mg/kg, daily) or DMSO as control by intragastric administration. After treatment for 8 days, tumor weight and size were measured, tumor tissues were further proceeded for PCR or western blot analysis.

### RNA sequencing

GISTs from 8-week-old KIT^V558A/WT^ mice and KIT^V558A/WT^/ZSWIM4^−/−^ mice were frozen in liquid nitrogen, total RNA was isolated and sequenced using Illumina NovaSeq6000 sequencing platform (Biomarker Technologies Corporation, Beijing, China). Raw data in FASTQ format was aligned with the reference genome of *Mus musculus* (GRCm38) by STAR (v2.7.9a). Transcript abundances were quantified by RSEM (v1.3.3) to estimate the expression level of genes. DESeq2 (v1.36.0) was utilized to detect differentially expressed genes (DEGs) at |fold-change|≥ 1.5 and adjusted p < 0.05. Obtained DEGs were then input for gene ontology (GO) analysis by clusterProfiler (v4.7.1). RNA sequencing data have been deposited in SRA database (PRJNA1068295).

### Statistical analysis

The results of three independent experiments were presented as mean ± standard deviation. The statistical difference was calculated using GraphPad Prism software. p < 0.05 was considered as statistically significant (*p < 0.05, **p < 0.01, ***p < 0.001, ****p < 0.0001).

## Results

### ZSWIM4 is expressed in GISTs

**Fig. 1:**
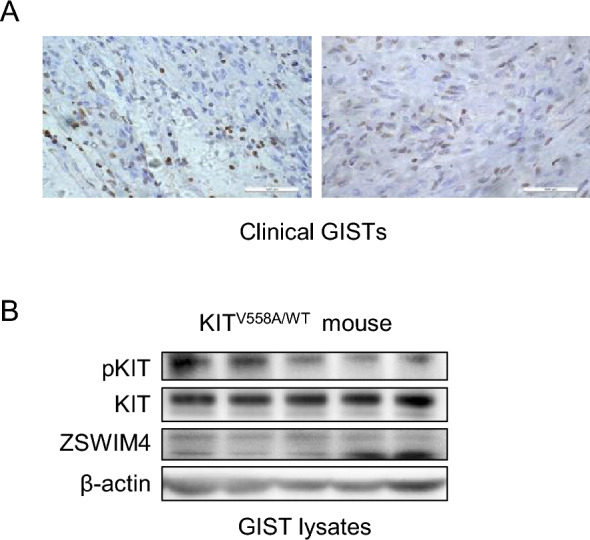
ZSWIM4 expression in GISTs. **A** ZSWIM4 expression in clinical GISTs was examined by immunohistochemistry with two cases as representative.** B** ZSWIM4 expression in GISTs of KIT^V558A/WT^ mice was examined by western blot

ZSWIM4 is a zinc finger protein with its knowledge pretty limited. To investigate its potential involvement in GISTs, we began by assessing ZSWIM4 expression in clinical GIST samples. Immunohistochemistry results showed that ZSWIM4 is expressed in GISTs (Fig. 1A). This finding was corroborated by western blot analysis of GISTs from KIT^V558A/WT^ mice (Fig. 1B), further suggesting the expression of ZSWIM4 in GISTs.

### KIT inhibits ZSWIM4 expression through PI3 kinase/AKT while ZSWIM4 inhibits KIT expression and downstream signaling in GISTs

**Fig. 2:**
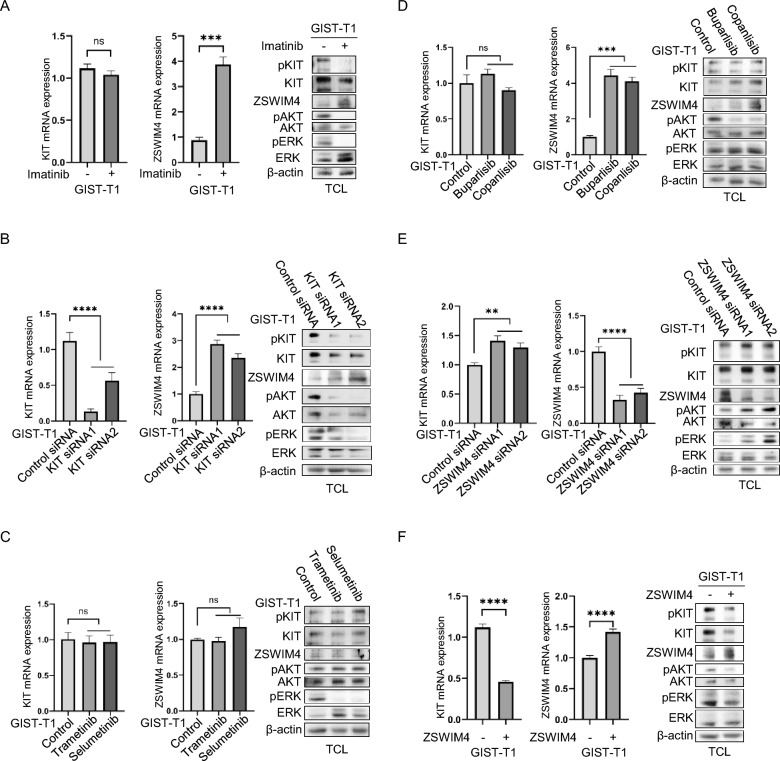
KIT inhibits ZSWIM4 expression while ZSWIM4 inhibits KIT expression and downstream signaling in GISTs. **A** GIST-T1 cells were incubated with DMSO or imatinib (1 μM) for 8 h, the expression of KIT and ZSWIM4 was examined by qRT-PCR. Cells were lysed and total cell lysates (TCL) were probed with 4G10, KIT, ZSWIM4, pAKT, AKT, pERK, ERK and β-actin antibodies respectively. **B** GIST-T1 cells were transfected with control siRNA or KIT siRNAs, the mRNA expression of KIT and ZSWIM4 was examined by qRT-PCR, and the total cell lysates were examined by western blot as indicated. **C** GIST-T1 cells were treated with MEK inhibitors trametinib and selumetinib respectively, the mRNA expression of KIT and ZSWIM4 was examined by qRT-PCR, and the total cell lysates were examined by western blot as indicated.** D** GIST-T1 cells were treated with PI3 kinase inhibitors buparlisib and copanlisib respectively, the mRNA expression of KIT and ZSWIM4 was examined by qRT-PCR, and the total cell lysates were examined by western blot as indicated.** E** GIST-T1 cells were transfected with control siRNA or ZSWIM4 siRNAs, the mRNA expression of KIT and ZSWIM4 was examined by qRT-PCR, and the total cell lysates were examined by western blot as indicated. **F** GIST-T1 cells were transfected with control plasmid or ZSWIM4 expressing plasmid, the mRNA expression of KIT and ZSWIM4 was examined by qRT-PCR, and the total cell lysates were examined by western blot as indicated

KIT mutations account for 70–80% of GISTs with KIT signaling being pivotal in their tumorigenesis [6–9]. To investigate whether KIT signaling regulates ZSWIM4 expression in GISTs, GIST cells were treated with imatinib or KIT siRNAs respectively to inhibit KIT activation or expression. Subsequent analysis by quantitative RT-PCR and western blot revealed that both imatinib and KIT siRNAs upregulate ZSWIM4 expression (Fig. 2A, B), suggesting that KIT signaling inhibits ZSWIM4 transcription in GISTs. PI3 kinase/AKT and RAS/RAF/MEK/ERK signaling pathways play an important role in KIT signaling [20, 21, 27], in order to know whether the two signaling pathways are involved in the regulation of ZSWIM4 expression by KIT, GIST-T1 cells were treated with PI3 kinase inhibitors or MEK inhibitors. Both qRT-PCR and western blot results showed that PI3 kinase inhibitors, but not MEK inhibitors, increase ZSWIM4 expression (Fig. 2C, D), meaning that activation of PI3 kinase/AKT signaling pathway inhibits ZSWIM4 expression. Considering that the activation of PI3 kinase/AKT is dependent on KIT (Fig. 2A), the results suggested that KIT inhibits ZSWIM4 expression through PI3 kinase/AKT signaling pathway.

In addition to the regulation of ZSWIM4 expression by KIT, we found that knockdown of ZSWIM4 expression or overexpression of ZSWIM4 respectively increases or decreases KIT expression in GIST cells by qRT-PCR and western blot (Fig. 2E, F). This bidirectional regulation suggests that KIT and ZSWIM4 participate in a negative feedback loop. Moreover, alteration in ZSWIM4 expression influenced the activity of AKT and ERK, downstream effectors of KIT. Specifically, knockdown of ZSWIM4 expression resulted in increased AKT and ERK activation, whereas ZSWIM4 overexpression had the opposite effect (Fig. 2E, F). These findings imply that ZSWIM4 acts as a suppressor of KIT signaling in GISTs.

### ZSWIM4 inhibits GIST cell survival and proliferation in vitro

**Fig. 3:**
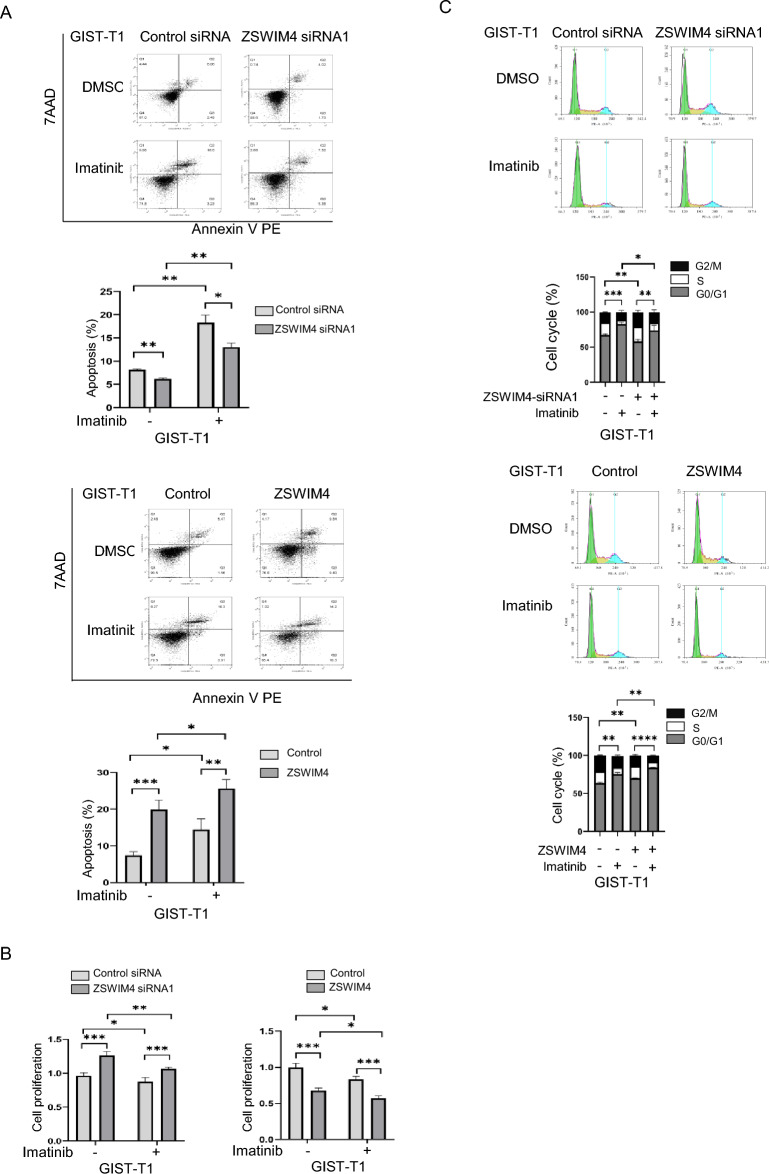
ZSWIM4 inhibits GIST cell survival and proliferation in vitro. **A** GIST-T1 cells were transfected with control siRNA or ZSWIM4 siRNAs, vector control or ZSWIM4 expressing plasmid, treated with DMSO or imatinib (0.0125 μM). After incubation for 72 h, cell survival was examined by flow cytometry after staining using PE Annexin V Apoptosis Detection Kit. **B** Cell proliferation was examined by MTT assay. **C** Cell cycle progression was examined by flow cytometry after staining with PI

Given that ZSWIM4 suppresses KIT expression and its downstream signaling, we further examined the role of ZSWIM4 in GIST cell survival, proliferation and cell cycle progression in vitro. Our findings indicated that knockdown of ZSWIM4 expression enhances GIST cell survival, while ZSWIM4 overexpression has a diminishing effect (Fig. 3A). In line with this, knockdown of ZSWIM4 expression led to increased cell proliferation and cell cycle progression, while overexpression resulted in the opposite outcomes (Fig. 3B, C). These results support ZSWIM4's role as an inhibitor of GIST cell growth, consistent with its negative modulation of KIT signaling.

To explore the functional relationship between ZSWIM4 and KIT, the expression of KIT and ZSWIM4 was knocked down. The results showed that knockdown of KIT expression, or both KIT and ZSWIM4 expression inhibit cell proliferation to a similar level (supplementary Fig. 1A), and knockdown of ZSWIM4 expression didn’t show any additional effect, probably due to the fact that KIT is dominant in GISTs, and which is supported by that almost all GIST cells are killed by imatinib at high concentration (data not shown). To further explore the impact of ZSWIM4 on the response of GISTs to imatinib, which is used as the first-line targeted therapeutic drug of GISTs and its treatment efficiency is dependent on the tumor's response to the drug, the GIST cells were treated with a low concentration of imatinib. The results showed that alterations in ZSWIM4 expression does not affect the cells' sensitivity to imatinib, as the drug effectively inhibited GIST cell survival and proliferation irrespective of ZSWIM4 levels although knockdown of ZSWIM4 expression and ZSWIM4 overexpression respectively increased or decreased cell survival, proliferation and cell cycle progression (Fig. 3A, B, C). In addition, we didn’t see any alteration of cell proliferation in the presence of DMSO at the concentration of 0.0125%, which is the highest concentration of DMSO in cell proliferation when used as solvent for imatinib, while the lowest concentration of DMSO is 3.2% to inhibit GIST-T1 cell proliferation (supplementary Fig. 1B).

### ZSWIM4 downregulates BMAL1 expression to inhibit GIST cell survival and proliferation

**Fig. 4:**
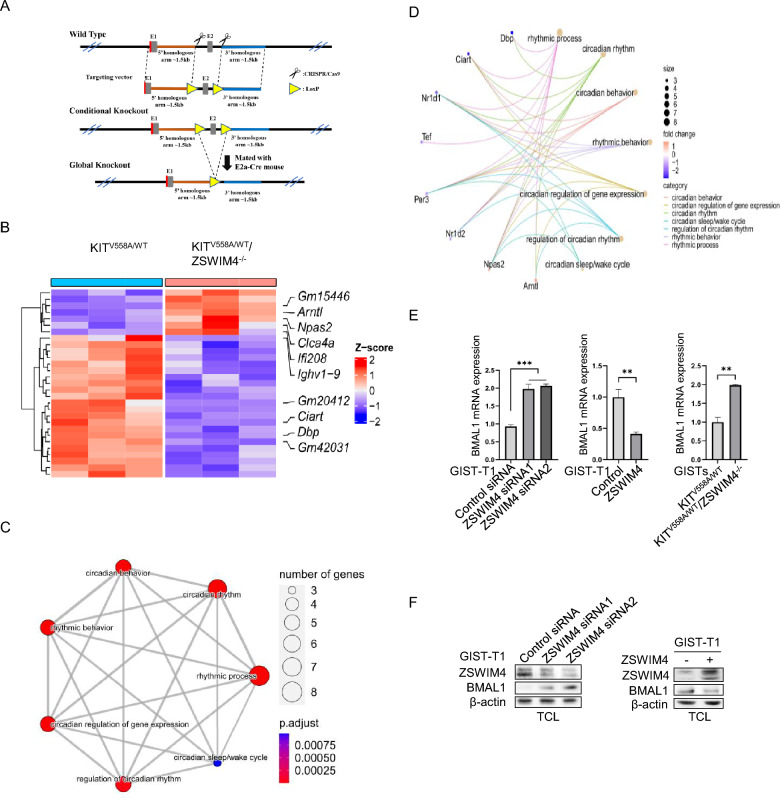

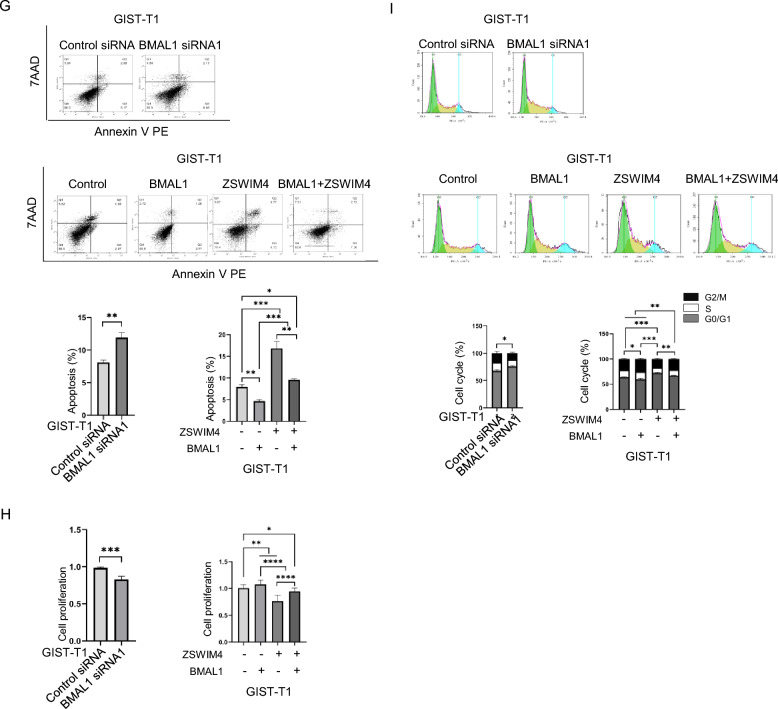
ZSWIM4 decreases BMAL1 expression to inhibit GIST cell survival and proliferation. **A** The construction of ZSWIM4-/- mice. **B** Heatmap of all identified DEGs in GISTs between 8-weeks-old KIT^V558A/WT^ mice and KIT^V558A/WT^/ZSWIM4^−/−^ mice (N = 3) after RNA sequencing using Illumina NovaSeq6000 sequencing platform. Top 5 upregulated and downregulated DEGs are labelled respectively. **C** Results of GO analysis show top 7 enriched GO terms. GO terms with shared genes are interconnected. **D** Changes of gene expression in enriched GO terms of rhythmic process. Positive fold-change indicates upregulation in ZSWIM4 KO group and vice versa. **E** GIST-T1 cells were transfected with control siRNA or ZSWIM4 siRNAs, vector control or ZSWIM4 expressing plasmid, total RNA was extracted from the cells or GIST tissues of 8-week-old KIT^V558A/WT^ mice and KIT^V558A/WT^/ZSWIM4^−/−^ mice, the mRNA expression of BMAL1 was examined by qRT-PCR. **F** GIST-T1 cells were transfected with control siRNA or ZSWIM4 siRNA, control plasmid or ZSWIM4 expressing plasmid, and total cell lysates were probed with ZSWIM4, BMAL1 and β-actin antibodies respectively. **G** GIST-T1 cells were transfected with control siRNA or BMAL1 siRNA, vector control, BMAL1 expressing plasmid, ZSWIM4 expressing plasmid, or both ZSWIM4 and BMAL1 expressing plasmids, and cell survival was examined by flow cytometry after staining using a PE Annexin V Apoptosis Detection Kit. **H** Cell proliferation was examined by MTT assay. **I** Cell cycle progression was examined by flow cytometry after staining with PI

To further understand the mechanism by which ZSWIM4 regulates GISTs beyond its inhibition on KIT signaling, we created ZSWIM4 knockout (ZSWIM4^−/−^) mice (Fig. 4A). These mice were crossed with KIT^V558A/WT^ mice to establish KIT^V558A/WT^/ZSWIM4^−/−^ mice. Upon performing RNA sequencing on GISTs from both KIT^V558A/WT^ and KIT^V558A/WT^/ZSWIM4^−/−^ mice, 29 DEGs were identified, in which 22 DEGs are downregulated in GISTs from KIT^V558A/WT^/ZSWIM4^−/−^ mice (Fig. 4B). Top 7 enriched GO terms are related to rhythmic processes and circadian behavior (Fig. 4C), suggesting that ZSWIM4 regulates the expression of genes involved in circadian rhythm. Within the GO terms associated with the rhythmic processes, we have identified 8 DEGs being regulated (Fig. 4D). To verify the RNA sequencing results, the expression of circadian clock pathway members in GISTs from KIT^V558A/WT^ mice and KIT^V558A/WT^/ZSWIM4^−/−^ mice were analyzed by qRT-PCR. The results showed that the mRNA expression of BMAL1 (ARNTL), NPAS2 is increased while the expression of TEF, NR1D2, NR1D1, and DBP is decreased when ZSWIM4 expression is knocked out (Fig. 4E, Supplementary Fig. 1C), which agrees with the RNA sequencing results. Further investigation into ZSWIM4's role in circadian clock regulation was conducted by either knocking down or overexpressing ZSWIM4 in GIST-T1 cells. Consistent with the qRT-PCR analysis of gene expression in GISTs from KIT^V558A/WT^ mice and KIT^V558A/WT^/ZSWIM4^−/−^ mice, ZSWIM4 inhibits the mRNA expression of circadian clock pathway members BMAL1 and NPAS2 but promotes the expression of TEF, NR1D2, NR1D1, and DBP (Fig. 4E, Supplementary Fig. 1D, E). In addition, western blot results showed that knockdown of ZSWIM4 expression or overexpression of ZSWIM4 respectively increases or decreases BMAL1 expression in GIST cells (Fig. 4F).

It has been reported before that the circadian clock pathway is involved in cancer in addition to its key role in the regulation of circadian rhythm [28, 29], while the circadian clock pathway member BMAL1 can act as either a tumor suppressor or oncogene in different malignancies [29–31]. In our results, we found that knockdown of BMAL1 expression or overexpression of BMAL1 respectively decreases or increases GIST cell survival, proliferation and cell cycle progression, while overexpression of BMAL1 and ZSWIM4 simultaneously reduces the inhibition of cell survival, proliferation and cell cycle progression by ZSWIM4 (Fig. 4G, H, I), suggesting that BMAL1 contributes to GIST cell survival and proliferation, and ZSWIM4 may inhibit GIST cell survival and proliferation through inhibition of BMAL1 expression.

### KIT increases the distribution of ZSWIM4 in the nucleus

**Fig. 5:**
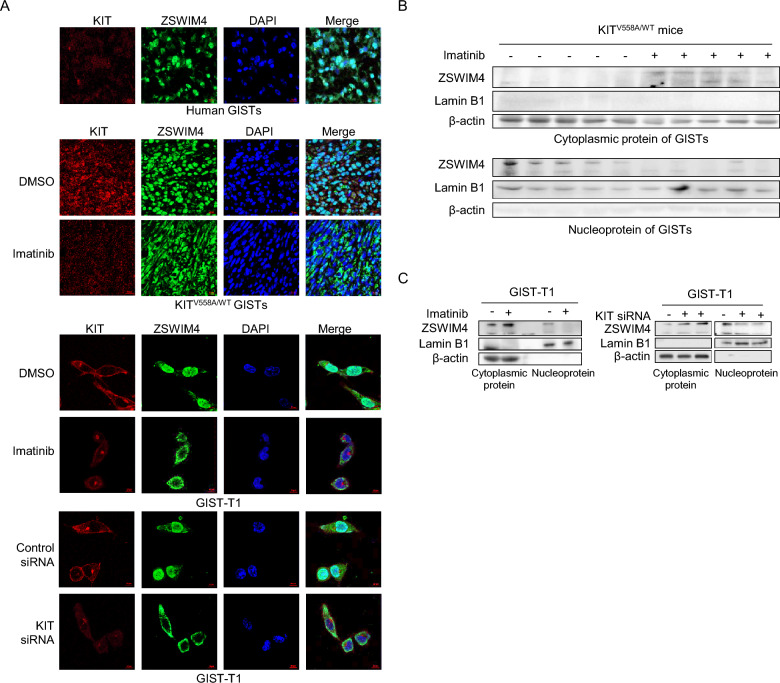
KIT increases the distribution of ZSWIM4 in the nucleus. **A** Subcellular localization of ZSWIM4 and KIT in clinical GISTs, murine GISTs (8-week-old KIT^V558A/WT^ mice were treated with DMSO or 50 mg/kg imatinib daily for 8 days) and GIST-T1 cells (treated with DMSO or imatinib, control siRNA or KIT siRNA) was examined by immunofluorescence (630 ×). **B** 8-week-old KIT^V558A/WT^ mice were treated with DMSO or imatinib (50 mg/kg daily for 8 days), and the cytoplasmic protein and nucleoprotein were extracted from GISTs and probed with ZSWIM4, Lamin B1, and β-actin antibodies respectively.** C** GIST-T1 cells were treated with DMSO or imatinib (1 μM), or transfected with control siRNA and KIT siRNA, cytoplasmic protein and nucleoprotein were extracted and probed with ZSWIM4, Lamin B1, and β-actin antibodies respectively

In our recent study, we found that ZSWIM4 is expressed in the nucleus of the Spemann-Mangold organizer in *Xenopus* embryos [23]. To further understand the function of ZSWIM4 in GISTs, we studied the subcellular localization of ZSWIM4 in GISTs. Immunofluorescence examination of clinical GISTs showed that ZSWIM4 is localized in both cytoplasm and nucleus with nucleus predominant (Fig. 5A), which was further confirmed in the examination of GISTs from KIT^V558A/WT^ mice and GIST-T1 cells by immunofluorescence examination and western blot after cell fraction (Fig. 5A, B, C). However, treatment of KIT^V558A/WT^ mice with imatinib, and treatment of GIST cells with imatinib or KIT siRNAs increase the distribution of ZSWIM4 in the cytoplasm (Fig. 5A, B, C), indicating that KIT signaling is important for the entry of ZSWIM4 to the nucleus.

### The entry of ZSWIM4 to the nucleus is important for its inhibitory role in GIST cells

**Fig. 6:**
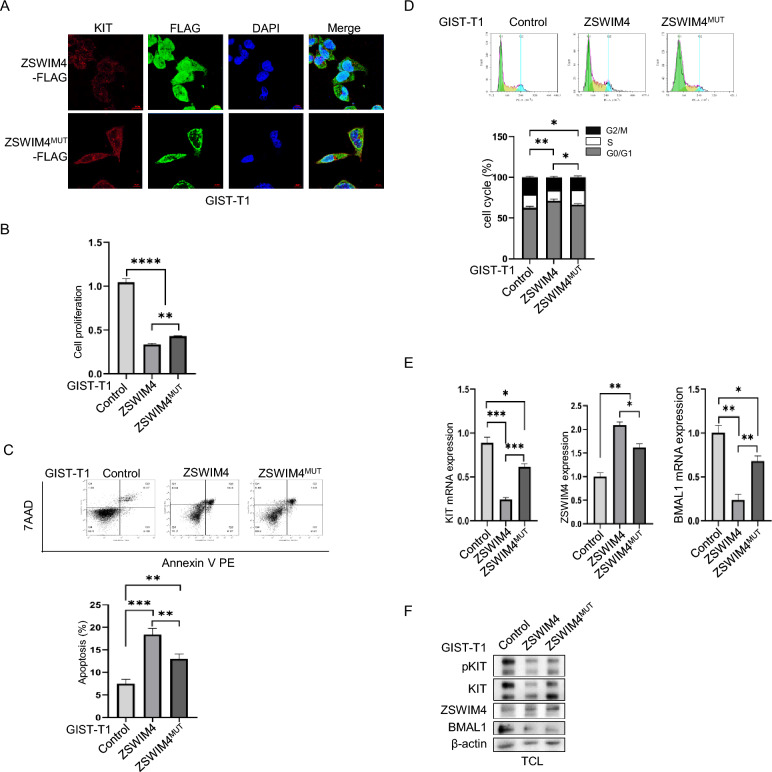
The entry of ZSWIM4 to the nucleus is important for its inhibitory role in GIST cells. **A** Subcellular localization of ZSWIM4 and KIT in GIST-T1 cells transfected with ZSWIM4 expressing plasmid or ZSWIM4^MUT^ expressing plasmid (FLAG tagged) which lacks 45 amino acids in the N-terminal. **B** GIST-T1 cells were transfected with control plasmid, ZSWIM4 expressing plasmid, ZSWIM4^MUT^ expressing plasmid, after incubation for 72 h, cell proliferation was examined by MTT assay. **C** Cell survival was examined by flow cytometry after stained using PE Annexin V Apoptosis Detection Kit. **D** Cell cycle progression as examined by flow cytometry after stained with PI. **E** GIST-T1 cells were transfected with control plasmid, ZSWIM4 expressing plasmid, ZSWIM4^MUT^ expressing plasmid, mRNA expression of KIT, ZSWIM4, and BMAL1 were examined by qRT-PCR. **F** The protein expression of KIT, ZSWIM4 and BMAL1 were examined by western blot

To further investigate the significance of ZSWIM4 localization within the nucleus of GIST cells, we engineered a mutant ZSWIM4 construct that lacks N-terminal 45 amino acids which are necessary for its entry to the cell nucleus (Fig. 6A). The results showed that overexpression of both wild-type ZSWIM4 and ZSWIM4^MUT^ inhibit GIST cell survival, proliferation and cell cycle progression. However, the inhibitory effects were less pronounced with ZSWIM4^MUT^ compared to wild-type ZSWIM4 (Fig. 6B, C, D). This observation suggests that the entry of ZSWIM4 to the nucleus is important for its inhibition of GIST cell survival and proliferation. Consistent with these findings, overexpression of both wild-type ZSWIM4 and ZSWIM4^MUT^ inhibited the expression of both KIT and BMAL1 with the wild-type ZSWIM4 exerting a stronger inhibitory effect than ZSWIM4^MUT^ (Fig. 6E, F). These results imply that the nuclear presence of ZSWIM4 is important for its role in downregulating key elements involved in GIST pathophysiology.

### ZSWIM4 inhibits the tumorigenesis of GISTs in vivo

**Fig. 7:**
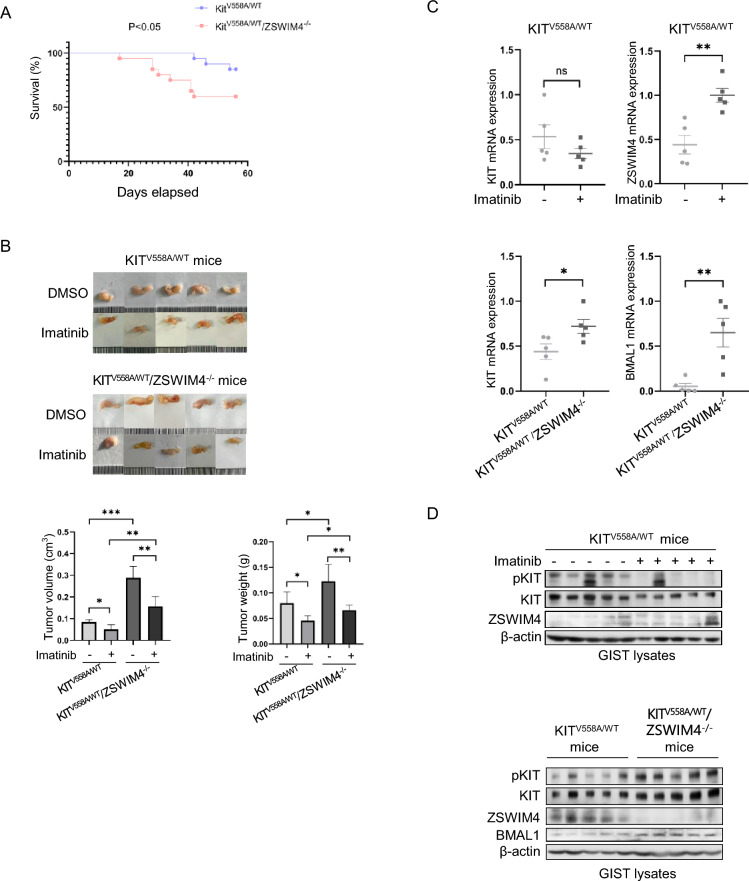
ZSWIM4 inhibits the tumorigenesis of GISTs in vivo. **A** The survival of KIT^V558A/WT^ mice and KIT^V558A/WT^/ZSWIM4^−/−^ mice (N = 20). **B** 8-week-old KIT^V558A/WT^ mice and KIT^V558A/WT^/ZSWIM4^−/−^ mice were treated with DMSO or imatinib (50 mg/kg daily for 8 days), tumor weights and tumor volumes were measured. **C** Total RNA was extracted from GISTs, the mRNA expression of KIT, ZSWIM4, and BMAL1 were examined by qRT-PCR. **D** Lysates of GISTs were probed with 4G10, KIT, ZSWIM4, BMAL1, and β-actin antibodies, respectively

Given its inhibitory effects observed in vitro, we conducted further research by knocking out ZSWIM4 expression in KIT^V558A/WT^ mice. We found that KIT^V558A/WT^/ZSWIM4^−/−^ mice survive shorter than KIT^V558A/WT^ mice (Fig. 7A), and experience accelerated GIST growth (Fig. 7B), suggesting that ZSWIM4 inhibits the tumorigenesis of GISTs in vivo. Moreover, after treatment with imatinib, tumor volumes in KIT^V558A/WT^ mice and KIT^V558A/WT^/ZSWIM4^−/−^ mice were reduced by 40.6% and 45.7% respectively, and tumor weight was reduced by 42.9% and 46.1% respectively, suggesting that ZSWIM4 does not influence the sensitivity of GISTs to imatinib treatment. This is consistent with in vitro findings that ZSWIM4 does not alter GIST cell sensitivity to imatinib, despite its inhibitory role (Fig. 3A, B, C). Additionally, examination of tumor tissues from KIT^V558A/WT^ mice and KIT^V558A/WT^/ZSWIM4^−/−^ mice by qRT-PCR and western blot showed that loss of ZSWIM4 expression increases KIT and BMAL1 expression (Fig. 7C, D). This is in agreement with the in vitro results (Figs. 2C, D and 4C, D), reinforcing the notion that ZSWIM4 inhibits the tumorigenesis of GISTs through its inhibition of KIT and BMAL1 expression. In addition, imatinib treatment of KIT^V558A/WT^ mice increased ZSWIM4 expression in GISTs, which agrees with the results in vitro as well (Fig. 2A). Taken together, these results suggested that ZSWIM4 inhibits KIT and BMAL1 to suppress the tumorigenesis of GISTs.

## Discussion

GISTs are most commonly driven by KIT mutations [6–9], making KIT a primary therapeutic target for the disease. Although four lines of targeted therapeutic drugs have been used in clinic and they have dramatically improved the treatment outcomes of GISTs [10, 17–19], however, drug resistance to KIT inhibitors remains a challenge, with some patients experiencing treatment failure. Addressing this issue requires a deeper understanding of KIT signaling regulation to improve therapeutic strategies. In this study, we investigated the role of the zinc finger protein ZSWIM4 in GISTs, and our results suggested that ZSWIM4 serves as a tumor suppressor in GISTs by inhibition of KIT signaling. In addition, ZSWIM4 expression is inhibited by KIT signaling. Therefore, KIT and ZSWIM4 form a negative feedback loop in GISTs. KIT signaling is tightly regulated by ligand binding and KIT activation-mediated ubiquitination and degradation of KIT in normal tissues [21, 22, 32], while the oncogenic mutations lead to ligand-independent activation of KIT in GISTs, and SCF facilitates an autocrine/paracrine loop that exacerbates the oncogenic process [33]. In addition, our results discovered a novel regulatory mechanism of KIT signaling in GISTs, which further increases our understanding of the regulation of KIT signaling in GISTs.

So far, ZSWIM4 has not been extensively studied, and our current understanding of its functions is quite limited. Previous research has indicated that ZSWIM4 reduces the sensitivity of breast cancer cells to JAK2 inhibitor [34], and ZSWIM4 might play a role in the response to vitamin D treatment in colon tissues [35], although the underlying mechanism is not elucidated yet. We recently found that ZSWIM4 regulates embryonic patterning by complexed with the Cul2-RING ubiquitin ligases, ELOB and ELOC, to promote the ubiquitination and degradation of SMAD1 [23]. Similar to ZSWIM4, the other members of the zinc finger SWIM-domain containing family were not widely studied as well although distinct functions have been showed between them. In alcohol injured liver, ZSWIM3 inhibits inflammation by binding with tumor necrosis factor receptor-associated factor 2 to mediate the activation of the nuclear transcription factor κB (NF-κB) pathway in macrophages [36]. ZSWIM6 is associated with acromelic frontonasal dysostosis [37] and neuronal development [38, 39]. ZSWIM7 is associated with ovarian insufficiency [40, 41]. ZSWIM8 is involved in mediating microRNA degradation [42]. In this study, we found that ZSWIM4 inhibits the tumorigenesis of GISTs, and the localization of ZSWIM4 in the nucleus is necessary for its inhibition on KIT signaling and circadian clock pathway member BMAL1 in GISTs. These results further increase our knowledge about ZSWIM4. However, further research is needed to elucidate the detailed mechanisms governing the subcellular localization of ZSWIM4 and its regulatory effects on KIT signaling and the circadian clock pathway.

The circadian clock is a complex mechanism that synchronizes cellular activities and coordinates the actions of different tissues and organs to form an approximate 24-h oscillation through the transcription-translation feedback loop [43, 44]. Beyond their role in regulating these rhythms, components of the circadian clock pathway have also been implicated in cancer, although their functions vary across different malignancies [28, 45]. For example, the core circadian pathway members PER2 and BMAL1 serve as tumor suppressor in lung cancer [30], BMAL1 and CLOCK respectively act as tumor suppressor and oncogene in colorectal cancer [31], BMAL1 inhibits ferroptosis to increase the oncogenesis of acute myeloid leukemia [29]. While our results showed that BMAL1 contributes to the tumorigenesis of GISTs. This suggested that BMAL1 may have cellular context-dependent functions, underlining the complexity of the circadian clock components in cancer biology.

## Conclusion

Taken together, our results suggested a negative feedback loop between KIT and ZSWIM4 in GISTs, where ZSWIM4 serves as a tumor suppressor in GISTs by inhibiting KIT signaling and BMAL1, a key component of the circadian clock pathway. These results strengthen our understanding of the regulation of KIT signaling and the tumorigenesis of GISTs, providing a stronger theoretical foundation for the development of more effective GIST treatments.

### Supplementary Information

Supplementary material 1. A. Cell proliferation of GIST-T1 cells transfected with control siRNA, ZSWIM4 siRNA, KIT siRNA, or ZSWIM4 siRNA plus KIT siRNA by MTT assay. B. Cell proliferation of GIST-T1 cells in the presence of DMSO by MTT assay. C. mRNA expression of genes in GISTs of KITV558A/WT mice and KITV558A/WT/ZSWIM4-/- mice was examined by qRT-PCR. D. mRNA expression of genes in GIST-T1 cells transfected with control siRNA or ZSWIM4 siRNAs was examined by qRT-PCR. E. mRNA expression of genes in GIST-T1 cells transfected with control plasmid or ZSWIM4 expressing plasmid was examined by qRT-PCR.

## Data Availability

The data supporting the current study are available from the corresponding author upon reasonable request.
