# Supplementary figures and images for: Entry of ZSWIM4 to the nucleus is crucial for its inhibition of KIT and BMAL1 in gastrointestinal stromal tumors
Source: Cell Biosci. 2024 Jun 29;14:87. doi: 10.1186/s13578-024-01271-z (PMC11218225; doi:10.1186/s13578-024-01271-z)

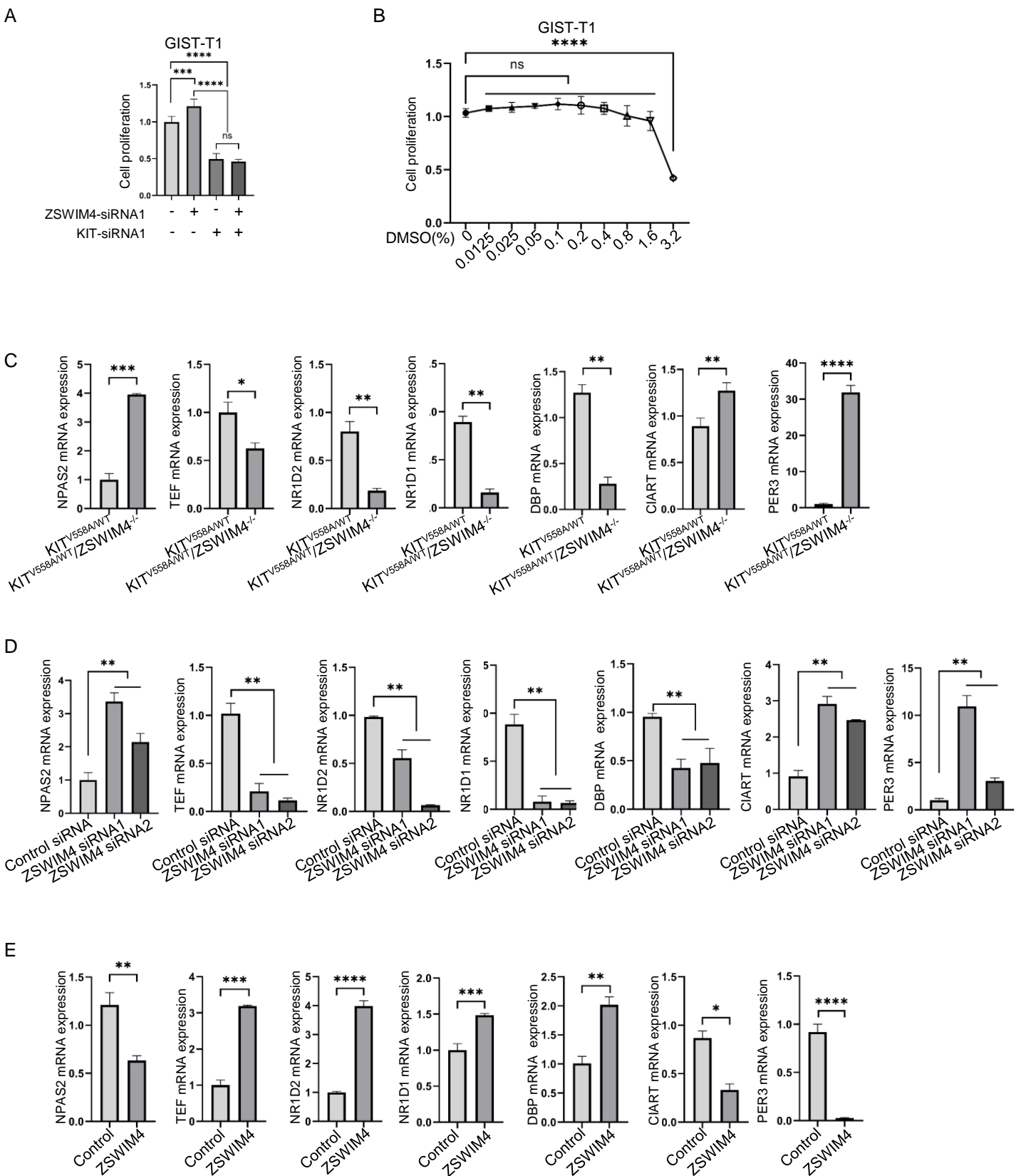

Supplement: Supplementary file 1 — Supplementary material 1. A. Cell proliferation of GIST-T1 cells transfected with control siRNA, ZSWIM4 siRNA, KIT siRNA, or ZSWIM4 siRNA plus KIT siRNA by MTT assay. B. Cell proliferation of GIST-T1 cells in the presence of DMSO by MTT assay. C. mRNA expression of genes in GISTs of KITV558A/WT mice and KITV558A/WT/ZSWIM4-/- mice was examined by qRT-PCR. D. mRNA expression of genes in GIST-T1 cells transfected with control siRNA or ZSWIM4 siRNAs was examined by qRT-PCR. E. mRNA expression of genes in GIST-T1 cells transfected with control plasmid or ZSWIM4 expressing plasmid was examined by qRT-PCR. [file 13578_2024_1271_MOESM1_ESM.pdf]
